# Resilience testing in action – piloting the health system resilience testing tool with a pandemic scenario in Finland

**DOI:** 10.1186/s12913-025-12864-w

**Published:** 2025-06-03

**Authors:** Liina-Kaisa Tynkkynen, Soila Karreinen, Markku Satokangas, Marjaana Viita-aho, Ilmo Keskimäki, Julia Zimmermann, Philip Haywood, Jonathan Cylus, Marina Karanikolos

**Affiliations:** 1 Welfare State Research, Finnish Institute for Health and Welfare, Helsinki, Finland; 2 Faculty of Social Sciences, Health Sciences, Tampere University, Tampere, Finland; 3 Faculty of Medicine, Department of General Practice and Primary Health Care, University of Helsinki, Helsinki, Finland; 4 European Observatory on Health Systems and Policies, London School of Economics and Political Science, London, UK; 5 Leeder Centre for Health Policy, Economics, and Data, University of Sydney, Sydney, Australia; 6 European Observatory on Health Systems and Policies, London School of Hygiene and Tropical Medicine, London, UK

**Keywords:** Health system resilience, Resilience testing, Health system performance assessment, Pandemic preparedness

## Abstract

**Background:**

System-wide approaches to measure, prepare for and manage the next acute shock are needed. We document the application of the health system resilience testing tool to a hypothetical pandemic scenario in Finland.

**Methods:**

The resilience testing tool promoted pre-crisis identification of resilience gaps and was built on the Health Systems Performance Assessment Framework and the Shock Cycle Framework. It included guidance on building a shock scenario, conducting a semi-structured resilience testing dialogue with health system stakeholders, and evaluating resilience.

A hypothetical scenario of a pandemic affecting predominantly children was addressed in a semi-structured, mixed-methods resilience test in Finland. The resilience test brought together national experts and other stakeholders to identify the health system weaknesses exposed by the scenario.

**Results:**

The resilience testing tool enabled the preparation for the high-level dialogue that identified actionable systemic weaknesses that undermine resilience. The identified weaknesses in the Finnish health system included: a lack of clarity of the process and value-basis of decision-making; sustaining trust towards and between authorities; multi-sectoral collaboration; safeguarding the health workforce; and developing a comprehensive knowledge base.

**Conclusions:**

The main benefit of the resilience testing methodology is the ability to bring key actors together to exchange different perspectives on how a health system functions during a crisis. The discussions at the high-level dialogue revealed the need for a mechanism, such as a resilience testing tool, to elucidate the range of practical challenges and how to potentially address them. The discussions also captured themes that are not routinely identified in existing performance assessment mechanisms, such as ethical considerations, values, and political determinants of the health system response. The Finnish pilot study was used to update the structure and facilitation of the resilience testing tool. Further suggested improvements for resilience testing include greater clarification for participants on the scenario, an increased emphasis on recovery and learning, and a greater representation of stakeholders from the community.

**Supplementary Information:**

The online version contains supplementary material available at 10.1186/s12913-025-12864-w.

## Background

Concern has grown that health systems are not well prepared to withstand acute shocks. Financial crisis, increased level of migration, the COVID-19 pandemic, heatwaves and other events have demonstrated the need for strategies that help health systems to be better prepared, mitigate, and learn from shocks [1–4]. Assessing resilience remains a challenging task due to the multifactorial nature of potential shocks and the complexity of health systems functioning [5].

Consequently, there has been a lack of methods to assess the resilience of health systems in a systematic way prior to crises. Existing methods have been criticised for putting too much emphasis on the measurable technological capacity of a health system and less so on the qualitative aspects of a successful response, such as organisational features and trust [6]. There has been a growing interest in resilience testing, drawn from experiences in other sectors such as banking, and the suggestion of “what if” scenarios to explore critical weaknesses in health system resilience [7–9].

Addressing these limitations, a testing methodology was produced by the European Observatory on Health Systems and Policies and the Organisation for Economic Co-operation and Development (OECD). This resilience testing tool was piloted in a one-day event (hereafter test day) in Finland in April 2023 using a pandemic scenario [10]. The handbook guided the preparations for the resilience testing dialogue, while the methodology was adapted to fit the Finnish context.

This article aims to outline the experience of applying the methodology and to discuss its advantages and limitations. We also summarise the key strengths and weaknesses of the Finnish health system that were identified during the resilience test day. The primary purpose of implementing the resilience test was to help operationalise a new instrument for policy making, not an academic exercise. Nonetheless, we think it important to share our learnings as a basis for possible future research.

## Methods

### Test methodology

The 'Strengthening Health Systems: A Practical Handbook for Resilience Testing' publication outlines a tool for testing the resilience of health systems [10]. The structured, systematic approach uses a shock scenario that aims to push the health system to breaking point to identify critical health system weaknesses that may only manifest in the case of such a shock. The scenario is to be chosen by test organisers based on health policy priorities and tailored to the country context. The analysis combines two existing frameworks. First, the global health system performance assessment (HSPA) framework provides a structure for the health system by outlining the key health system functions (i.e. governance, financing, resource generation and service delivery) [11]. Second, the shock cycle framework with four stages (preparedness, shock onset and alert, shock impact and management, recovery and learning) prompts consideration of the resilience of the health system both before, during and after the disruption caused by the hypothetical shock [1]. Health system resilience in the handbook has been defined as “[t]he capacity of a health system to a) proactively foresee, b) absorb, and c) adapt to shocks and structural changes in a way that allows it to i) sustain required operations, ii) resume optimal performance as quickly as possible, iii) transform its structure and functions to strengthen the systems and iv) (possibly) reduce its vulnerability to similar shocks and structural changes in future.” [10].

The preparatory step of the resilience testing consists of an assessment of the evidence on health system performance, focussed on the areas most affected by the shock. The assessment includes contextual information as well as an overview of selected HSPA indicators. The assessment guides facilitators of the resilience testing dialogue.

### The scenario

The tool was piloted using a scenario in which a globally spreading infectious disease that met the criteria of a pandemic caused a sudden rise in demand in health care service and threatened to overwhelm the health system. The pandemic scenario was developed by the experts from Finnish Institute for Health and Welfare (THL) in consultation with Ministry of Social Affairs and Health. The scenario was chosen because the consequences of the COVID-19 pandemic were still notable and there was an opportunity to inform discussion regarding health system recovery and resilience to future pandemics. At the time of the pilot, the Finnish health system had also recently undergone a major restructuring. From January 2023 responsibility for organising health care, social care and rescue services was transferred from municipality level (around 200 local authorities) to 22 newly formed regional-level entities - wellbeing services counties. At the same time, the health system funding model was also centralised with the state allocating funding to the counties based on a mainly needs based formula [12]. Consequently, the pilot study was carried out at a time when a large-scale structural reform had just taken place, and the new system was in the process of adjustment. Thus, the pilot study provided a good opportunity to discuss the strengths and weaknesses of health system resilience in the new Finnish structure.

The scenario was based on a pandemic triggered by a pathogen which had a strong impact on children between the ages of six months and seven years, causing substantially increased hospitalisations and mortality (see next section). It was considered that the health system response to the shock described in the scenario would be sufficiently different from that of the COVID-19 pandemic. Table 1 outlines the scenario.

**Table 1:** The outline of the pandemic scenario

What	Pandemic caused by a new infectious disease
Where	Finland
When	Long-term crisis / duration about 2 years
Why	Rapid global spread of the pathogen to a population with no previous immunity
Target population	The whole population, with young children and the elderly at risk for severe disease

In the scenario, a new disease was discovered outside Europe. After three weeks of detecting the pathogen the first cases were diagnosed simultaneously in several locations in Europe. The pathogen was identified as a quickly spreading new strain of virus, previously undetected in humans. The disease featured respiratory symptoms, a short incubation period and airborne transmission via mainly asymptomatic adults. Alongside young children, those who were overweight, with lung or heart disease, or aged over 70 years were at a high risk of serious complications. In the most severe cases, the disease would rapidly progress to severe breathlessness, which would require intensive care and prolonged respiratory support. Paediatric patients would usually require hospitalisation 1–2 weeks after onset, with mortality increasing after 3–4 weeks, resulting in a surge of patients. One of the first European cases was identified during a holiday season in the Northern Finland and subsequently the disease spread rapidly throughout the Finnish population in successive waves, causing the service system to become overburdened.

In the first phase of the pandemic scenario no vaccines or curative drugs were available. Therefore, only containment measures such as respiratory protection, physical distancing, enhanced ventilation of buildings and travel restrictions, as well as restrictions on private and public gatherings could be used to manage the epidemic. Controlling the spread of the disease, however, would be difficult. In the early stages of the pandemic, the primary mode of transmission of the virus would not be known, adults would present no symptoms, risk groups would not be reliably identified, and the significant number of patients would overwhelm healthcare capacity. Hospital admissions and mortality would rise sharply, especially in children’s wards and intensive care units. There would be many uncertainties about case fatality and the need for hospitalisation. A virus specific test was assumed to eventually become available during the first month of the pandemic, but testing capacity would still be limited, thus requiring prioritisation.

The scenario also outlined several other phenomena related to the onset of such a pandemic which would impact the health system’s capacity to cope and create disruptions in service delivery. These included a widespread fear of a communicable disease dangerous for children and with unknown long-term effects. Such fear would make parents less likely to take their children to day care or school and more likely to stay at home to care for their children. This would occur for many healthcare workers which, combined with the sick leave of infected healthcare workers, would lead to severe staff shortages and staff overburdening. The scenario suggested that strain on the staff would likely be high, as professionals without paediatric experience would also be needed in children’s units. Moreover, given the global nature of the outbreak, the supply chains for medicines, particularly those used to relieve symptoms in paediatric patients (e.g. paracetamol, ibuprofen, asthma inhalers and antibiotics), personal protective equipment and other materials needed for paediatric care could be significantly disrupted, causing shortages of both medicines and supplies.

Viewed through the framework developed for assessing the performance of the health system used in the resilience testing pilot, the scenario would cause an immediate disruption in the availability of resources (staff, materials, medicines) and in the production of services. In the longer term, a pandemic could lead to changes in legislation and thus affect the wider health system structures. Additional funding would also be needed.

### Constructing the scenario and designing the resilience test day in Finland

The scenario for the resilience dialogue was developed in consultation with the experts from the health security unit at the Finnish Institute for Health and Welfare (THL), who have experience in building infectious diseases scenarios for tabletop exercises and simulations. For the scenario and the resilience test day the assessment was conducted according to the instructions and examples of the Handbook for Resilience Testing [10]. First, the resilience test organisers (the first five authors) briefly summarised research and grey literature on the topic and obtained national statistics for the current supply of services relevant to the scenario. Next, the organisers outlined how the scenario might impact the Finnish health system and what (and at what level of detail) should be included in the materials compiled for the participants. The materials were intended to focus on the resilience dialogue and avoid any side-tracking to “solving” the scenario.

Finally, the organisers went through an iterative process of producing questions inspired by the examples in the handbook to guide the discussions on the test day. The organisers categorised each question according to the HSPA framework and the different stages of the shock cycle [1, 11]. The questions were workshopped with the developers of the methodology from the European Observatory on Health Systems and Policies and the OECD. Then the resilience test organisers selected the final questions that were considered the most relevant for the Finnish context.

Participants of the resilience test day were invited to represent key organisations and institutions involved in Finnish pandemic governance. The choice was based on appropriateness of their role in pandemic response and to ensure representation of stakeholders. The identification was carried out in collaboration with the Ministry of Social Affairs and Health. A personal email invitation was sent to 27 experts, 21 of whom registered to participate, with 18 attending the resilience test day. The participants represented different levels and administrative branches, e.g. the Ministry of Social Affairs and Health, the Ministry of Education and Culture, the Prime Minister’s Office, Ombudsman for children, the wellbeing services counties (regional authorities responsible for the provision and financing of health, social and rescue services), municipalities and the university hospitals.

The participants received the background materials which included the scenario and a short description of the HSPA framework in advance of the resilience test day via email. The key points of the circulated material were also presented at the beginning of the test day by the resilience test organisers. The pilot day discussions were chaired by the organisers who are experienced health systems and policy researchers, and notes were taken by three researchers from the THL. The participants signed a consent form giving permission to report the results of the pilot on a general level without their identity being revealed.

**Fig. 1:**
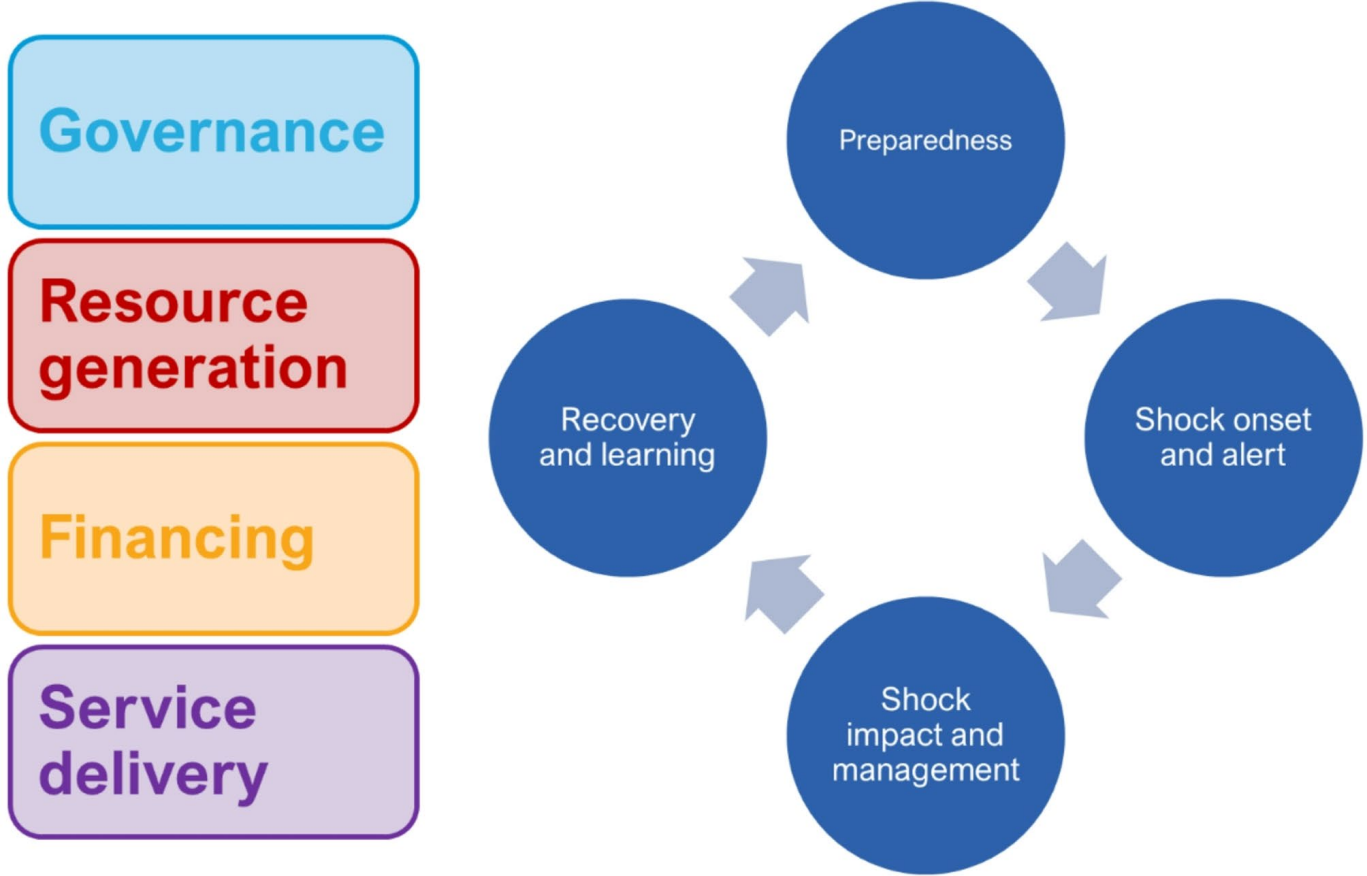
Health system functions and the shock cycle

The health system functions were further explored through structured discussion by a group of relevant stakeholders. The discussion was structured around the shock cycle (preparedness, onset and alert, impact and management, and recovery and learning), within which the questions focussed on health systems functioning were addressed (see Fig. 1 and the list of questions in the Additional file 1).

It was emphasised to participants that the aim of the test day was not to “solve the scenario” as in a tabletop exercise, but to consider the resilience of different health system functions from varying perspectives at each stage of the shock cycle. Results were collected through taking notes during the discussion and through an anonymous online voting process where participants were asked to identify the top strengths and weaknesses of the health system for each stage of the shock cycle and for the overall scenario.

The resilience test day was divided into four sets of discussions according to the four stages of the shock cycle. However, the stages three (shock impact and management) and four (recovery and learning) were eventually combined to facilitate the flow of the discussion.

The discussions were facilitated by using the me-we-us method [13]. Each discussion was guided by questions, which the participants first contemplated by themselves for a couple of minutes, after which the questions were discussed in small groups (see Additional file 1). Group notetaking in a web-based platform was also supported to identify key strengths and weaknesses of the health system in regard to the scenario. After each group discussion all the participants were engaged in a round table discussion for sharing and elaborating their conclusions on different health system functions and aspects of resilience. Voting on strengths and weaknesses of the health system occurred after each discussion using the same web-based platform as in group notetaking.

Representatives from the European Commission, OECD and the European Observatory on Health Systems and Policies observed the resilience dialogue. The discussions were simultaneously interpreted for the non-Finnish participants.

### Evaluating the resilience test implementation

To evaluate the resilience testing methodology, a mixed-methods approach was used. This approach included direct observation of the resilience test day by the test developers, a semi-structured interview with the organisers and facilitators of the resilience test, and a short online post-test day feedback questionnaire for the participants of the resilience test (See Additional file 2).

## Results

### Evaluating the resilience test and its implementation

The resilience test day resulted in informative discussions around the set of prepared questions, with further elaboration on implications of the scenario for specific aspects of health system functioning. The experiences from the recent COVID-19 pandemic were often cited, as was to be expected given the scenario was closely related. The resilience testing tool helped the facilitators to guide the participants to systematically consider the relevant health system aspects. Discussions in smaller groups allowed for optimal use of time and consensus building. The round table discussions concluding each stage provided a shared understanding of the implications of the scenario, in which some participants were more active than others. The participants’ varying levels of engagement in round table discussions emphasised the importance of complementing such arrangements with alternatives. In the Finnish case, breakout sessions for small groups with rapporteur reporting and anonymous voting allowed all participants to express their thoughts in different ways.

Based on the feedback received from twelve out of eighteen participants, the resilience testing pilot was deemed to have been a very valuable and positive experience. The participants pointed out that the wide-ranging conversation was especially useful and provided new insights and some clarity for many of them on the topic of health system resilience. The background materials and the scenario sent to the participants the day before the pilot day were appreciated. Some inconsistencies in the scenario, however, were identified by the participants. Participants also noted the need for more clarity to the focus of the testing. Some participants wished for more time to familiarise themselves with the material before the pilot day, noting that the material was extensive. The participants assessed the pilot day to have been well facilitated and offered them good opportunities to contribute to the discussions.

Improvements for future exercises were suggested. One suggestion was that reflections on the outcomes during the day should be developed to ensure they accurately capture the conversation. This may, for instance, include asking the participants to validate or challenge the points raised by the facilitators. Another suggestion was that additional stakeholders from the community should be invited to participate, such as communications specialists, social sector experts, representatives of the media and politicians.

### Evaluating the Finnish health system resilience in the light of a pandemic scenario

#### Health system preparedness

For the preparedness stage, the participants raised concerns about the inflexibility of the legislative frameworks which did not, in 2023, enable agile reactions to acute and wide-ranging crises requiring multisectoral collaboration. Thus, it was suggested that amendments should be made to current legislation, especially the Communicable Diseases Act and the Act on Organising Healthcare and Social Welfare Services, in line with previous findings based on the experiences of the COVID-19 health system response [14]. The discussions emphasised that more attention should be paid to planning the recruitment of additional personnel and building and utilising the varying competencies of different health system professionals. In the discussion, the high proportion of specialists in the healthcare sector was noted as a possible barrier for adaptation in times of crisis when people would need to assume tasks outside their scope of expertise. This ability should be fostered and supporting knowledge, skills and mental wellbeing were recognised as key to sustaining a crisis-responsive healthcare workforce. Participants discussed that the healthcare workforce should be allowed to participate in preparedness planning, which had not been the case previously in Finland. In addition to securing adequate levels of health professionals, the sufficiency of hospital beds in a long-lasting crisis was identified as a challenge to be addressed both nationally and regionally. The participants also suggested that horizontal and vertical cross-sectoral collaboration should be supported.

#### Onset and alert

When assessing the Finnish health system’s capacity to identify the onset of a crises and managing the alert phase of the shock cycle the participants underlined the importance of national-level priority setting. Their view was that prioritisation would most probably begin on the local and regional level. To ensure the equity of services nation-wide, national steering and planning of prioritisation should support these lower-level decisions. Higher-level directions would also provide backup support for an individual civil servant in the very probable climate of conflicting value emphases and even harsh counter-reactions. However, regional differences (such as geographical, demographical, and service network issues) should be taken into consideration. The participants suggested that the hospital system’s capacity to deal with the pandemic could double by reducing elective care, but only for a limited time. As in the preparedness stage, cross-sectoral collaboration was recognised as crucial to guaranteeing efficient mitigation measures and securing the overall well-being of the population. The requirement for collecting and sharing information from different sources between stakeholders was highlighted by the participants. Again, collaboration across administrations, as well as between sectors and geographical regions, was called for to consolidate these efforts. Consequently, legislation should support information gathering and sharing. Communication to the public was recognised as an important and delicate matter: transparency was regarded as crucial in the face of a new pathogen, but “public panic” should be avoided. The uncertainty of the situation and information flows from non-official sources were identified as challenges.

#### Impact and management

Due to the scenario, impact and management of the pandemic would be of a long-lasting nature. Therefore, ensuring provision of essential health services was deemed crucial by the participants. This would need balancing between acute and elective care, possibly supported by digital solutions. Administrative siloes and sectoral distribution of financing may challenge collaborative endeavours. It was widely agreed that funding in the acute phase of the crisis would not as such develop into a problematic issue. Instead, difficulties could emerge from decisions on how to balance the funding of different sectors (health and social sectors, school, day care etc.) and between acute and non-acute services. In times of crisis, it was noted that it may be easier and more appealing to enforce strict measures that show immediate benefits even if they have long-term disadvantages. The participants recognised that decision-making during a pandemic is challenging, especially when the situation requires extensive restrictions. There was a discussion about developing legislation that contains provisions that are only operational when certain conditions are met, anticipating the needs during crises. In addition to gaining agility in governing the constantly changing pandemic, some participants reasoned that this could help balance the challenges of valuing different impacts, such as the relationship between human rights and healthcare needs. Participants argued that making shorter-term decisions on restriction measures might prevent potential harm caused by them. It was also suggested that regular revision of decisions should be regarded as an integral part of pandemic management measures, and it could reduce the risk of exaggerated responses.

#### Recovery and learning

The recovery stage was deemed important to strike the balance between the crises and the “normal” state, and to clarify the end of the acute phase and the beginning of the recovery. On the one hand, “ending a crisis” meant adjusting from certain ways of operating and prioritising new functions (e.g. scaling back testing and vaccination capacity). On the other hand, the end of a crisis challenged the stakeholders to recognise what ways of working would be beneficial to maintain during normal times. It was noted that deliberation and learning take time, which is often limited.

The discussion revealed that the recovery phase is often overlooked and thus often lacks political attention and sufficient resources. Reflecting on both the impact and management of the crisis as well as recovering and learning from it, the participants concluded that the major challenges of Finnish health system resilience relate to how, by whom and on what grounds difficult choices (requiring balancing between different values) are made. It was considered that political decision-makers should be responsible for making these choices and communicating them. It was also noted that financial support may diminish in the recovery stage, making it more challenging to identify and implement lessons learned.

## Discussion

Despite the widely recognised need to strengthen health system resilience, the operationalisation of the resilience concept and the existence of practical tools to assess health system resilience to a wide range of shocks remain very limited [5]. In this article, we document experiences from applying the health system resilience testing tool using a pandemic scenario to the context of a national health system in Finland [10].

Although Finland has robust pandemic preparedness plans, the COVID-19 pandemic showed that comprehensive assessment of risks and input from a range of stakeholders into policy advice is still lacking [14–16]. Therefore, piloting the resilience testing dialogue was seen by the Ministry of Social Affairs and Health and the Finnish Institute for Health and Welfare as a potentially useful mechanism to strengthen pandemic preparedness and evaluate the lessons learned from the COVID-19 pandemic from the policy-makers’ perspective. At the time of the test day the national pandemic plan was being updated. As part of the health and social services reform, five new regional authorities, centres for preparedness in healthcare and social welfare were established in Finland. Their role as part of the network of authorities is still being established, but their main role will be to form, maintain and share a situation picture of the healthcare and social welfare service system, covering the entire primary healthcare, specialised healthcare, prehospital emergency medical care and social welfare [17, 18].

The tool was tested using a pandemic as a shock scenario, which was purposefully close to the recent COVID-19 pandemic yet differed in terms of the key population affected (young children). The rationale of dealing with a scenario featuring a pandemic enhanced relatability and relevance - participants’ experience of COVID-19 enriched the discussion and made it realistic. Relating the pandemic scenario to a different population group (children as opposed to those aged over 80 or with chronic conditions [19]) enabled participants to approach the exercise with a fresh view and focus. This meant that the resilience test day provided an opportunity for many participants to reflect on the lived experiences that are applicable to wider pandemic contexts jointly with experts from different sectors and levels of government.

The Finnish pilot was welcomed by the invited participants and a group of key stakeholders present on the resilience test day. One of the key benefits of the pilot listed by both the participants and the organisers was that it provided a forum to bring the key actors together and to address different perspectives in a joint discussion. Despite Finland being a country with high level of transparency and stakeholder engagement [19] the feedback from the test day showed that many participants found it was a rare opportunity to engage in such a multidisciplinary discussion. Participants pointed out that hearing the views of different actors was valuable and helped to understand the practical aspects of pandemic responses to be implemented.

Therefore, a key benefit of resilience testing is the potential to bring actors together and discuss different perspectives. This can have a resilience enhancing effect, as it gives the key decision makers an opportunity to better understand the perspectives of those involved in crises. The tool can also help draw attention to the complex nature of the health system functioning during crises.

The Finnish pilot study contributed substantially to the development of guidance on the structure and facilitation of the resilience test day published in the Resilience Testing Handbook [10]. The pilot study identified challenges in feasibility if the resilience test extends beyond one day, introduced the “me-we-us” facilitation technique and digital voting tools. These lessons contributed to the development of guidance for future organisers of resilience testing including recommendations on the number of participants, the structure of the resilience testing workshop and the methods for capturing participants’ views. The pilot reinforced the structuring of discussions according to the stages of the shock cycle. The Finnish pilot demonstrated, in practice, that grounding resilience testing in HSPA and shock cycle frameworks ensures systematic approach to identifying strengths and weaknesses. While the scenario guides the conversation to certain aspects of health systems responses, it may not fully capture all the essential areas of resilience. However, applying health system functions, sub-functions and assessment areas outlined in the HSPA Framework over shock cycle stages reveals broader challenges and sub-optimal health system functioning that may be common to multiple shocks [10]. For completeness, a series of resilience testing exercises with different scenarios could provide further insights into other specific areas of action that may be more relevant in different contexts.

The main practical challenge in this pilot was related to framing the approach to identify key strengths and weaknesses of a certain health system instead of a simulation exercise in which the goal is to find a solution to the scenario. This difference should be very clearly and repeatedly communicated to the participants, allowing them to grasp the idea of focussing on identifying systemic problems and policy solutions rather than addressing technical processes. When conducted after a crisis, resilience testing can also serve as a forum to draw lessons from the experiences and supporting both intra-crisis and inter-crisis learning [20]. In the Finnish case, it also highlighted the impact of changes brought about with the recent reform that focussed on the centralisation of health care to the wellbeing services counties [12].

The participants of the resilience test day noted that the learning and recovery stage received little attention on the day. This was criticised by participants on the basis that this was the same pattern as in their working environment. This is consistent with other findings concerning the governance of the COVID-19 pandemic in Finland, according to which the window for gathering lessons learned in the public and political discussion was limited [21].

The resilience test day highlighted that health system resilience cannot be considered as a technical system characteristic or objective that can be measured and achieved only by strengthening core system functions such as governance, financing, resource generation or service delivery. In the discussion, certain aspects of governance, in particular values, ideologies, power structures, and politics, emerged as key elements that contribute to determining how pandemics are managed and on whom the burden of the direct and indirect effects of a pandemic fall (e.g. vulnerable people). In the research literature, resilience has also increasingly come to be seen as a phenomenon which is always constructed or not constructed in the national and local contexts and in relation to other systems and actors [6]. Thus, health system preparedness and crisis management should be more sensitive to the interdependencies between different systems and their subsystems in local, national, and global contexts [22–24]. Health system resilience is also a highly political and value-laden issue, where different options and their impacts must be weighed up, possibly with limited information and under tight deadlines. Identifying values, politics, and power relations at the early stages of a shock can therefore help to take them into account during crises. The pilot showed that preparedness should also pay attention to issues that are often not explicitly part of the core functions of health systems or are not directly derived from the HSPA framework.

## Conclusions

The pilot demonstrated that testing of health system resilience using a semi-structured approach was feasible and has good potential to identify health system weaknesses that can be acted upon to strengthen health system resilience. Resilience testing could be a relevant and accessible approach for policy makers that combines evidence and experience by bringing together stakeholders that are at the forefront of a health system’s response to a shock. The resilience test day can be used to systematically address specific health system functions and sub-functions that come under stress during a shock, by facilitating a semi-structured discussion tailored to assessing the shock response. Identifying stakeholders that are best placed to participate in the discussion is a crucial element of successful resilience testing. The main benefit identified by participants was the opportunity to engage in a joint debate that highlighted multiple perspectives. It is likely that the same perspectives would be presented in the context of scenarios other than a pandemic, which was chosen as the case for the resilience test day piloted in Finland. The health system performance assessment framework and the shock cycle were used to systematically identify areas for investigation, but the participants suggested that systems that intersect with the health system were also important for health system resilience.

## Supplementary Information

Additional file 1. List of questions assigned to the small groups for discussion.

Additional file 2. Feedback form for pilot testing participants.

## Data Availability

The resilience testing methodology is publicly available. A more detailed report of the pilot day (in Finnish only) is available upon request.

## Abbreviations

HSPA: Health System Performance Assessment
OECD: Organisation for Economic Co-operation and Development
THL: Finnish Institute for Health and Welfare
