# Supplementary material for: Resilience testing in action – piloting the health system resilience testing tool with a pandemic scenario in Finland
Source: BMC Health Serv Res. 2025 Jun 3;25:793. doi: 10.1186/s12913-025-12864-w (PMC12135546; doi:10.1186/s12913-025-12864-w)
Supplement: Supplementary file 1 — Additional file 1. List of questions assigned to the small groups for discussion. [file 12913_2025_12864_MOESM1_ESM.docx]

# **Questions assigned to the small groups for discussion**

# **Preparedness**

| Governance | |
| --- | --- |
|  | - What capacity does the system have for cross-sectoral cooperation in the context of a widespread communicable disease? |
|  |  |
|  | - Do the current regulations give the authorities and professionals sufficient powers to act against a widespread infectious disease? |

| Resource generation | |
| --- | --- |
|  | - How would you evaluate the health system’s key vulnerabilities related to the health workforce? Consider, for example, whether the system has sufficient expertise to respond to the pandemic described in the scenario. - What preparations have been made for a situation in which professionals should be quickly trained to care for children? |
|  | - Are ventilators (suitable for the children) and other medical equipment geographically equitably distributed?  How about hospital beds? |
|  | - How would you evaluate the sufficiency of the mandatory reserve supplies and stockpiling for paediatric patients, both pharmaceuticals and other consumables? |

| Financing | |
| --- | --- |
|  | - How does the funding model for wellbeing services counties take preparedness into account, what are the incentives? |

# **Onset and Alert**

| Governance | |
| --- | --- |
|  | - How would you evaluate the health system’s capacity/ability to recognize a spreading, new communicable disease? Is there variation in the capacity across the country? - Is there a mechanism for organising data collection for achieving a full picture of the situation? - How quickly can testing capacity be scaled up? Who is responsible for that? |
|  | - Is it clear who has the responsibility to 1) declare an onset of a crisis, 2) decide on the implementation of planned crisis protocols? Are the responsibilities clear and do you consider them to be sufficient? What are the key vulnerabilities? - Who will take the lead/responsibility to coordinate the pandemic response at the national level? Is it clear and who decides on that? - How multisectoral coordination is initiated and what kind of structures there are in place for that? Can you identify any vulnerabilities in them? (Is it easy to identify the key actors? |

| Resource generation | |
| --- | --- |
|  | - Are there mechanisms to prepare the workforce for deployments? How will the staff transitioning to children’s wards and intensive care units be supported (ethical and emotional load may be significantly increased for example by having to make decisions on intensive care for children or meeting a sick children patient and their families)? |
|  | - How would you evaluate the health system’s ability to quickly increase paediatric ward and ICU capacity? How about testing capacity? - How would you evaluate health system’s ability to increase the supply/import/export of equipment, drugs and consumables needed to care for paediatric patients (e.g., redistributing products within Finland, or acquisition of new equipment from abroad)? What happens if considerable disruptions in the supply are experienced? |

| Service delivery | |
| --- | --- |
|  | - How will different actors in the health system and at different levels gain the information of the onset of the epidemic? - How are service prioritisation decisions made? How are health care professionals and decision-makers supported in these decisions? |

# **Impact and Management**

| Governance | |
| --- | --- |
|  | - How are the social impacts and impacts of pandemic response measures on different population groups assessed? - How is the social impact and impact of pandemic response measures on different population groups assessed? - Are there obvious gaps in the availability of routinely collected data? - With whom should the actors in the health system cooperate to ensure that children's rights to, for example, safe schooling and early childhood education are implemented as well as possible in the context of this scenario? |
|  | - How is it made sure that multisectoral collaboration is maintained and managed in a way that leads to an effective response? Can you identify any vulnerabilities, which might lead to inequitable response – i.e., that some vulnerable population groups are left outside the response? |

| Resource Generation | |
| --- | --- |
|  | - Are the emergency stockpiles for pharmaceuticals adequate in content and number in this scenario? Are there processes to ensure successful purchases when refilling the depleted stockpiles after the onset / in a prolonged crisis? (Either national plans or EU-level agreements? Who coordinates national/EU-level collaboration in these purchases?) - Are there mechanisms to ensure the availability of consumables? |

| Service delivery | |
| --- | --- |
|  | - In the context of that scenario, how can the rest of the service system be maintained? Which parts or functions of the system are more vulnerable to resource or workforce shortages when a shock such as the scenario occurs? How can these be addressed? |

| Financing | |
| --- | --- |
|  | - How is it made sure that the financial resources are sufficient and that funds are allocated equally and efficiently? |

# **Recovery and Learning**

| Governance | |
| --- | --- |
|  | - Is it possible/easy to identify who has missed out on appointments or surgeries in health care? What kind of other data are needed to design recovery from the pandemic? - Is there a process for systematically reviewing the learnings after major shocks and making necessary adjustments for the future? How are the experiences of the children and families collected? |

| Resource generation | | |
| --- | --- | --- |
|  | | - Are there plans in place on how health workforce returns to original tasks? What are the triggers to start shifting resources back to their usual allocations? - Are there mechanisms to support health workforce in returning to their own work? |

| Service delivery | |
| --- | --- |
|  | - How is it made sure that the practices designed for crisis are not unnecessarily maintained after the acute phase of the pandemic? |

| Financing | |
| --- | --- |
|  | - How can it be ensured that additional funding is allocated to deal with the consequences of the pandemic? How is the level and allocation of funding assessed? |
