# Supplementary material for: Resilience testing in action – piloting the health system resilience testing tool with a pandemic scenario in Finland
Source: BMC Health Serv Res. 2025 Jun 3;25:793. doi: 10.1186/s12913-025-12864-w (PMC12135546; doi:10.1186/s12913-025-12864-w)
Supplement: Supplementary file 2 — Additional file 2. Feedback form for pilot testing participants. [file 12913_2025_12864_MOESM2_ESM.docx]

**Health Systems Resilience Testing Pilot – Participant Feedback**

This feedback form will be used to gather feedback on the Health Systems Resilience Testing Pilot and to inform adjustments to the approach and methodology. These questions should be answered by individuals who participated in the Health Systems Resilience Test.

**Preparation and Communications**

1. Were the purpose and objectives of the Health Systems Resilience Test clearly communicated to you in advance? Please select one box.

|  |  |  |  |  |
| --- | --- | --- | --- | --- |
| 1 Not at All | 2 | 3 | 4 | 5 Very Clear |

2. Were the background materials sent to you in advance clear and easy to understand? Please select one box.

|  |  |  |  |  |
| --- | --- | --- | --- | --- |
| 1 Not at All | 2 | 3 | 4 | 5 Very Clear |

3. Did the background materials sent to you in advance prepare you to participate in the Health Systems Resilience Test? Please select one box.

|  |  |  |  |  |
| --- | --- | --- | --- | --- |
| 1 Not at All | 2 | 3 | 4 | 5 Very Well |

4. Was the shock scenario appropriate and relevant? Please select one box.

|  |  |  |  |  |
| --- | --- | --- | --- | --- |
| 1 Not relevant | 2 | 3 | 4 | 5 Very relevant |

5. How much time did you spend looking at materials/preparing for the pilot before the test day?

|  |
| --- |

6. Please provide any general comments on the **communications and materials** that you received in advance of the Health Systems Resilience Test.

|  |
| --- |

**Delivery**

7. Was the Health Systems Resilience Test facilitated in a clear and logical manner? Please select one box.

|  |  |  |  |  |
| --- | --- | --- | --- | --- |
| 1 Not logical | 2 | 3 | 4 | 5 Very logical |

8: Did you feel empowered to contribute to the exercise?

|  |  |  |  |  |
| --- | --- | --- | --- | --- |
| 1 Did not feel empowered | 2 | 3 | 4 | 5 Felt very empowered |

9. Did you have sufficient time to run through the exercise and consider the resilience of your health system in response to the shock scenario? Please select one box.

|  |  |
| --- | --- |
| Yes | No |

10. Did you have sufficient opportunities to contribute to the exercise? Please select one box.

|  |  |  |  |  |
| --- | --- | --- | --- | --- |
| 1 No opportunities | 2 | 3 | 4 | 5 Sufficient opportunities |

11. Were the visual tools used during facilitation useful in helping you to consider the resilience of your health system? Please select one box.

|  |  |  |  |  |
| --- | --- | --- | --- | --- |
| 1 Not at all | 2 | 3 | 4 | 5 Very useful |

12. Did you have sufficient knowledge about your health system to participate in and contribute to the Health Systems Resilience Test? Please select one box.

|  |  |  |  |  |
| --- | --- | --- | --- | --- |
| 1 Not enough knowledge | 2 | 3 | 4 | 5 Enough knowledge |

**Health Systems Resilience Testing Outcomes**

13. Were the test results an accurate reflection of the conversation during the Health Systems Resilience Test? Please select one box.

|  |  |  |  |  |
| --- | --- | --- | --- | --- |
| 1 Not accurate | 2 | 3 | 4 | 5 Very accurate |

14. Where the results of the day consistent with your expectations of the major weaknesses of the health system?

|  |  |
| --- | --- |
| Yes | No |

15. Was the Health Systems Resilience Test Pilot a valuable exercise for you? Please select one box.

|  |  |  |  |  |
| --- | --- | --- | --- | --- |
| 1 Not valuable | 2 | 3 | 4 | 5 Very valuable |

16. Do you think that results from the Health Systems Resilience Test will help you to identify and understand broader vulnerabilities in your health system (not specific to the shock scenario)?

|  |  |
| --- | --- |
| Yes | No |

17. Do you think you or the participants as a group might identify different strengths and weaknesses in the resilience of your health system if you tested it with a different shock scenario?

|  |  |
| --- | --- |
| Yes | No |

18. Are there any types of participants who did not attend the Health Systems Resilience Test who you think could have usefully participated?

|  |
| --- |

19. Please provide any comments on how the Health Systems Resilience Test has contributed to your thinking on Health Systems Resilience

|  |
| --- |

20. Please provide any general comments on the Health Systems Resilience Test and suggestions for improvement

|  |
| --- |
